# 

**Published:** 1897-09

**Authors:** 


### CONTENTS:

### SELECTIONS.

Article I.—Illinois State Dental Society, 1897 ..... 193
" II.—Some Points in the Ether-Chloroform Controversy ..... 209
" III.—Facts About Steel ..... 220
" IV.—Trichloracetic Acid as a Solvent of Necrosis. By H. W. Allwine, D. D. S ..... 222
" V.—Pyrozone. Conrad E. Wettlauffer, D. D. S ..... 225
" VI.—Discussion on Soldering ..... 227

### MONTHLY SUMMARY.

Pitiable Condition of a Girl from Use of X-Rays ..... 235

Filling Frail Teeth ..... 236

The Bacteriology of Baldness ..... 236

Root Perforation—A New Method of Treatment ..... 237

Rules for the Care of the Eyes ..... 238

Difficult Partial Impressions ..... 238

Suicide by Taking Cocain ..... 239

Cocain Solution ..... 239

Blood-poisoning after Tooth Extraction ..... 240

Nevermind the Other Fellow ..... 240

Different Method of Heating Guttapercha ..... 240

A Cure for Hiccough ..... 240

